# Formative research implications on design of a randomized controlled trial for oral health promotion in children

**DOI:** 10.1186/s40814-018-0344-y

**Published:** 2018-10-04

**Authors:** Molly A Martin, Helen H Lee, Jazmin Landa, Mark Minier, David Avenetti, Anna Sandoval

**Affiliations:** 1 Department of Pediatrics, University of Illinois at Chicago, 840 South Wood Street, Chicago, IL 60612 USA; 2 Department of Anesthesiology, University of Illinois at Chicago, 1740 West Taylor Street, Suite 3200, Chicago, IL 60612 USA; 3 Institute for Health Research and Policy, University of Illinois at Chicago, 1747 West Roosevelt Road, Room 558 M/C 275, Chicago, IL 60608 USA; 4 College of Dentistry, University of Illinois at Chicago, 801 S. Paulina, Chicago, IL 60612 USA

**Keywords:** Oral health, Children, Community health care, Qualitative research, Community health worker

## Abstract

**Background:**

The COordinated Oral health Promotion (CO-OP) Chicago trial will test the efficacy of a community health worker (CHW) intervention to improve oral health behaviors for children at high risk for early childhood caries. Before implementing the cluster-randomized controlled trial, we conducted a formative assessment to determine the final design. We used qualitative methods to assess the feasibility and acceptability of the proposed recruitment, data collection, and intervention plan.

**Methods:**

Key informant interviews (*N* = 37) and site observations were conducted at 10 pediatric primary care clinics and 10 Special Supplemental Nutrition Program for Women Infant and Children (WIC) centers to gain insight from providers and administrators at the locations where recruitment and intervention will occur. Eight focus groups (*N* = 68) were conducted with caregivers of children to capture the parent perspective. Conceptual coding methods from grounded theory were applied to organize the data into the final themes.

**Results:**

Families, clinics, and WIC centers were all very interested in additional supports for oral health and were enthusiastic about CHWs. Challenges included competing family priorities that might interfere in study enrollment and intervention efficacy. Physical space for enrollment and intervention delivery was a major barrier for some sites. Home visits for data collection and intervention delivery would be unacceptable for some families. These challenges and barriers prompted us to make major changes in our trial design. We opened the option for data collection to occur in multiple locations. We eliminated the home-only arm of the trial. Clinics and WIC centers that are randomized to the non-intervention arm will now have CHWs available at the study conclusion. Finally, we aligned the CHW oral health topics to the needs of families.

**Conclusions:**

We conducted this comprehensive formative assessment to determine the feasibility and acceptability of the CO-OP Chicago trial. While overall acceptance of the trial was high, the results highlighted specific issues with the proposed trial implementation plan and led to several critical design changes. This type of formative work requires a significant upfront investment but we expect it will translate into savings through better recruitment, retention, intervention implementation and adherence, and result dissemination.

**Electronic supplementary material:**

The online version of this article (10.1186/s40814-018-0344-y) contains supplementary material, which is available to authorized users.

## Background

Almost half of children 11 years old and younger have experienced dental caries [1]. Caries is one of the most common chronic diseases of childhood and is associated with pain, infections, malnutrition, speech difficulties, poor school performance, cosmetic problems, and an overall lower quality of life [2–4]. Low-income and minority children bear a disproportionate portion of this burden and its associated morbidity [2, 3, 5, 6]. Caries prevalence in Illinois and Chicago exceeds national figures, and Chicago area children have a high prevalence of untreated caries [7]. Many factors that contribute to caries in children are related to modifiable behaviors, including inadequate dental coverage and utilization, insufficient fluoride exposure, unhealthy dietary choices, and poor oral hygiene [8–10].

COordinated Oral health Promotion (CO-OP) Chicago is part of a National Institutes of Health health disparities research collaborative to develop and test interventions that aim to reduce pediatric oral health disparities. CO-OP Chicago proposes testing a community health worker (CHW) intervention that targets oral health behaviors for children at high risk for early childhood caries. CHWs are defined as frontline public health workers who serve as liaisons between health and social services and community residents [11]. CHWs typically provide health education, information, and assistance with services and build individual and community capacity for health [12]. Numerous studies support a role for CHW interventions in the improvement of health outcomes [13–16]. However, there are limited data on the ability of CHW intervention to improve child oral health outcomes or associated family behaviors [17–21].

While CHWs are frequently home-based, they also work out of clinical institutions and community agencies. CO-OP Chicago proposed placing CHWs in three types of locations: pediatric primary care clinics that serve low-income families; Special Supplemental Nutrition Program for Women, Infant, and Children (WIC) centers that are social service centers where families receive free formula and food; and homes. We were unsure how pediatric clinics and WIC centers prioritized oral health and how feasible the integration of research and CHWs would be into their sites. We also were not sure how families would react to this intervention and the associated research aims and methods. Therefore, before implementing the CO-OP oral health CHW intervention, we conducted a 2-year formative assessment to determine the final implementation structure for trial recruitment, data collection, and intervention delivery. The stakeholders included staff, providers, patient families, and clients at the clinics and WIC centers. We used a variety of methods to determine if stakeholders viewed oral health for young children as an important issue, if they were willing to support the activities (recruitment and intervention) of the project, and what barriers and opportunities they could foresee in regard to recruiting families, collecting data, and delivering an oral health CHW intervention in the sites and family homes.

We assessed the feasibility and acceptability of the proposed recruitment, data collection, and intervention plan using an approach from the field of design that applies grounded theory analysis to data from multiple different perspectives [22–24]. We obtained interview data from clinic and WIC center administrators, clinicians, and other staff (referred to as key informants), as well as focus group data from patients and clients using standard qualitative methods [24]. Sites were also observed to augment self-report with observational and contextual data [22, 23]. This manuscript describes that process and results and demonstrates the importance of conducting formative work before the implementation of behavioral trials.

## Methods

### Partner sites

We sought partnerships with medical clinics in Cook County, Illinois, that served primarily low-income Medicaid-insured patients. We started with the outpatient pediatric medical clinic affiliated with the University of Illinois at Chicago that serves a diverse, mainly lower-income patient demographic. The University is formally affiliated with a federally qualified healthcare center system that contains five pediatric clinic sites. A small private practice clinic near the University serving an exclusively Hispanic low-income population agreed to participate. Finally, another federally qualified healthcare system committed three clinical sites on the far south side of Cook County, Illinois.

WIC centers by definition serve low-income families. We partnered with two WIC agencies. Leadership from the Community Economic Development Association of Cook County, Inc., which supports a large number of local WIC centers, selected seven centers located in neighborhoods similar to the partner clinics. The Chicago Department of Public Health identified three additional WIC centers in separate neighborhoods.

### Human subjects

This study was approved by the University of Illinois at Chicago Institutional Review Board (Protocol # 2015-0815) and the Chicago Department of Public Health Institutional Review Board (#16-06). Participants provided written informed consent.

### Key informant interviews

Key informant interviews are in-depth interviews conducted with stakeholders who are experts in the topic being addressed [24]. The objective of the key informant interviews for this study was to gain insight into the feasibility and acceptability of the study design from the perspective of the clinics and WIC centers. Key informants were administrators, providers, and staff who could describe operations and clients in the clinics and WIC centers. Key informant recruitment used a purposive sampling strategy; site leadership recommended individuals who held the position of clinician, nurse, clinic administrator, or WIC center staff. Semi-structured interviews were conducted with key informants at their respective site by the study principal investigator and/or project manager with a research assistant. Interviews lasted 40–60 min and were audio recorded.

Key informants answered questions on demographics. We asked the key informants about the oral health strengths and challenges that they believed their patients/clients encounter as well as oral health challenges their site faced. To determine their interest in oral health and their likelihood of supporting our study initiatives, we asked about strategies their site has taken to try to help their patients/clients regarding oral health and interest they had in other oral health opportunities. To determine the feasibility and acceptability of our recruitment and data collection strategy, we described the plan to them and asked for their feedback. To determine the feasibility and acceptability of the CHW intervention, we asked their opinions on CHWs in general, important qualities of CHWs, and how to integrate CHWs into their clinic/WIC center. Finally, we asked what they would like to see in their site for incentives and outcomes. Key informants were offered $50 as an incentive.

From June 2016 to March 2017, we spoke with 37 individuals representing 20 sites. At the ten clinics, key informants included seven pediatric providers, six medical directors, four clinic managers, a clinic manager, a nurse manager, a medical assistant, and a clinical care coordinator. At the ten WIC centers, we spoke with four field coordinators, one nutritionist supervisor, six nutritionists, and five nutritionist technicians. At some sites, individuals held multiple roles such as clinic director and provider. Some administrators presided over more than one site. At three sites, we spoke to only one individual due to staffing changes. At the other 17 sites, we spoke to at least two individuals. Key informants were mainly female (86%). Thirty-five percent identified as White, 30% as Black, 16% as Asian, and 19% as Hispanic.

### Focus groups

Focus groups are guided discussions with groups of people regarding a specific topic or program [24]. Caregivers of children were convened in focus groups from December 2016 to March 2017 to provide the patient perspective regarding feasibility and acceptability of the study. To qualify, participants needed to be at least 18 years old, fluent in English or Spanish, and a primary caregiver of a child under the age of 3 (self-report) who was a patient or client in that clinic/WIC center. We selected 8 sites for recruitment that represented the main geographic locations and demographics of the 20 sites. Four of the eight focus groups were conducted in Spanish. We recruited between 21 and 31 potential participants per site from the waiting rooms and then sent reminders about the focus group date, time, and location. Most focus groups were held at the clinic/WIC center although two were held at public library community rooms near the clinic/WIC center when space was limited. The study principal investigator or project manager led the focus groups, which lasted 1.5–2 h. Participants answered questions on demographics and their caries history, and then we discussed their family’s experiences with oral health in order to determine their general interest in oral health. To determine the acceptability and feasibility of the study recruitment, data collection, and proposed intervention, we described the CO-OP study and asked caregivers to reflect on what they did and did not like about it, if they would participate, and how the study could be improved. Focus groups were audio recorded. Participants were offered $50 as an incentive.

In total, 68 caregivers (33% of those who expressed interest) participated in the focus groups. As shown in Table 1, most (95.6%) were female with a mean age of 31. The mean age of their children was 17.7 months (standard deviation of 16.2), although clinic caregivers had younger children than WIC caregivers (average 14.9 months and 20.3 months, respectively.) Sixty-two percent self-identified as Hispanic/Latino, 35% as Black, and 3% as White. The majority of children had Medicaid medical insurance (62%) or were unsure (37%, families frequently confuse the source of their insurance since the change to Medicaid managed care plans in Illinois), and most parents knew their children had dental insurance (84%). Many caregivers had no medical insurance (32%) and no dental insurance (36%). Seventy-one percent of caregivers reported having caries in the past.

**Table 1:** Focus group demographics

	8 sites, *N* = 68
Spanish language focus group (%)	35 (51.5)
Caregiver female (%)	65 (95.6)
Caregiver age, mean (SD)	30.9 (6.7)
Child female (%)	38 (55.9)
Child age in months, mean (SD)^a^	17.7 (16.2)
Child insurance (%)	
Medicaid	42 (61.8)
Private	0 (0.0)
Unsure type	25 (36.8)
None	1 (1.5)
Unsure if child’s medical insurance covers dental^b^ (%)	8 (11.9)
Child has dental insurance^b^ (%)	56 (83.6)
Caregiver insurance (%)	
Medicaid	26 (38.2)
Private	3 (4.4)
Unsure type	17 (25.0)
None	22 (32.4)
Caregiver has dental insurance^c^ (%)	38 (64.4)
Caregiver education (%)	
Less than high school	22 (32.4)
High school degree or GED	29 (42.7)
More than high school	17 (25.0)
Caregiver race (%)	
Black	24 (35.3)
White	2 (2.9)
Other	42 (61.8)
Caregiver Hispanic (%)	42 (61.8)
Mexican heritage^d^	29 (85.3)
Born in the mainland USA^e^	10 (23.8)
Caregiver relationship status (%)	
Married/living with partner	37 (54.4)
Single	29 (42.7)
Other	2 (2.9)
Total people living in home, median (range)	4 (2–10)
Number of children, median (range)	2 (1–8)
Caregiver has had caries in past (%)	48 (70.6)

^a^Although inclusion criteria during screening required a child under the age of 3, four caregivers in the WIC site sample reported a child age 4–7 years old. Because their data could not be separated out from the audio recordings, they were included in this table and analyses

^b^*N* = 67

^c^*N* = 59

^d^*N* = 34

^e^*N* = 42

### Site observations

Site observations provided data on the physical spaces and resources at the sites, as well as allowed for observations of persons in their natural settings which add an additional perspective to the assessment of study feasibility [22, 23]. These data were intended to verify or contradict the responses of the key informants and focus group participants. One research assistant observed the 20 sites once each from June 2016 to April 2017. Two sites were observed for 1.5 h, and the rest were observed for 2 h. The date and time were arranged with each site’s director, with the goal to capture a “typical” time. The research assistant sat in the waiting areas and observed the physical space and flow of people moving in and out. The research assistant did not engage with site staff or with patients. She took written notes to document how patients arrived (bus, car, foot), the check-in process (how staff greeted clients, wait times), when clients finished, the mood of clients and staff, and the decorations and furnishing (types of messages posted, television noise, play areas). If she was allowed to view other areas, their attributes were noted as well. The research assistant drew a map of each site and took pictures, when permitted, to capture the physical spaces. These maps highlighted entrances and exits, waiting areas, and private spaces where recruitment and intervention might be located.

### Analysis

Audio recordings were transcribed verbatim. Spanish transcripts were transcribed and analyzed in Spanish. (Research assistants translated selected quotes chosen for inclusion in an English language journal at the end of the analysis process.) The initial coding book was built from the interview/focus group guides and inputted into ATLAS.ti 7.5.16 Qualitative Data Analysis software (ATLAS.ti Scientific Software Development GmbH, Berlin, Germany). Two research assistants independently coded the first transcript. The original codebook was then modified to include new codes that emerged. Data was recoded and compared to verify concordance. Discrepancies were discussed, and another three transcripts were then double coded and compared. At this point, concordance was near 100%, and subsequent transcripts were single coded with close supervision. The second phase of grounded theory-based analysis consisted of conceptual coding [24]. For this phase, we used the query tool in ATLAS.ti to merge all the text associated with one particular code across all the transcripts. A team including the principal investigator, project manager, research assistants, and a co-investigator met to create conceptual categories. First, the key informant-coded data were reviewed. Data associated using the query tool were read together line by line by the team and then assigned to corresponding preliminary domains by group consensus. After reviewing all the data once, the team again reviewed and modified the preliminary domains to further distill the primary domains. The same process was done with focus group data. Then focus group and key informant primary domains were combined into the final domains by the team. During this process, areas of concordance and discordance were identified and emphasized. Lastly, the data from the site observations were reviewed by the team to identify areas where key informant and caregiver data could be verified, expanded, or contradicted. We looked at the notes, maps, and photos to identify spaces where recruitment, data collection, and intervention delivery could occur; we were assessing for visibility for recruitment and intervention delivery but also privacy for data collection. We examined the educational and client support materials on walls and shelves to determine interest in oral health and the target age group. These observations were incorporated to complete the final domain data.

## Results

### Acceptability of oral health

Families, clinics, and WIC centers were all very interested in additional supports for oral health. “I have one [a child] that is 18 years old and it happened to me in not knowing how to care for her teeth. And when I took her to the doctor she had lots of caries. I don’t want that to happen with her [another child].” (Hispanic female caregiver, Spanish) They discussed how the current system does not adequately meet the needs of families. “As I said before, in the dental office, I don’t know if they don’t have the time to educate the people.” (clinic key informant, medical director/provider)

### Acceptability of CHWs

Participants also overwhelmingly approved the primary CHW intervention. “…we dig community health workers around here.” (WIC center key informant, nutritionist); “…having a community worker who kind of knows how to coordinate that in trying to get them appointment and kind of get them plugged in, I think that would be beneficial.” (clinic key informant, pediatric provider); “Sometimes it feels better to have someone normal like you versus a doctor or someone telling you like okay you always saying that to everybody but if someone like us, you know, like, okay, you’re, you know, you feel like that’s a need too then maybe I’ll think that’s a need as well.” (African-American female caregiver) Preferred CHW qualities included similar race and language skills to the clients, ability to meet with families in a variety of clinical and community settings, good listening skills, empathy, and strong knowledge of oral health and community resources. We asked if CHWs needed to be a parent themselves in order to effectively support other parents, and it was determined that they did not.

### Feasibility challenges

Despite the interest in oral health, participants raised many concerns about priorities the families from these low-income communities face and how these issues might damper participation and the ability of families to make oral health behavior changes. “I don’t think they get the relationship between sugary liquids or sugary treats. Like you can say it and say it and say it. I think sometimes they just don’t get it, because we keep having kids – you know, the child is 3 and they’ve had all their teeth pulled and so I think that the message kind of blows over sometimes, because a lot of our clients have really big problems and not having that child cry sometimes can be the difference between being homeless on the street and having a place to stay at night, so I think our big problem is our parents are juggling much bigger problems…” (WIC center key informant, nutritionist); “Not even nutritional health, not even oral health, just survival is kind of a priority for some of our families.” (WIC center key informant, nutritionist) Parents and key informants expressed frustration about how difficult it was to make changes they knew were important. This was often related to multiple caregivers, unstable housing, and busy chaotic lives.

**Fig. 1:**
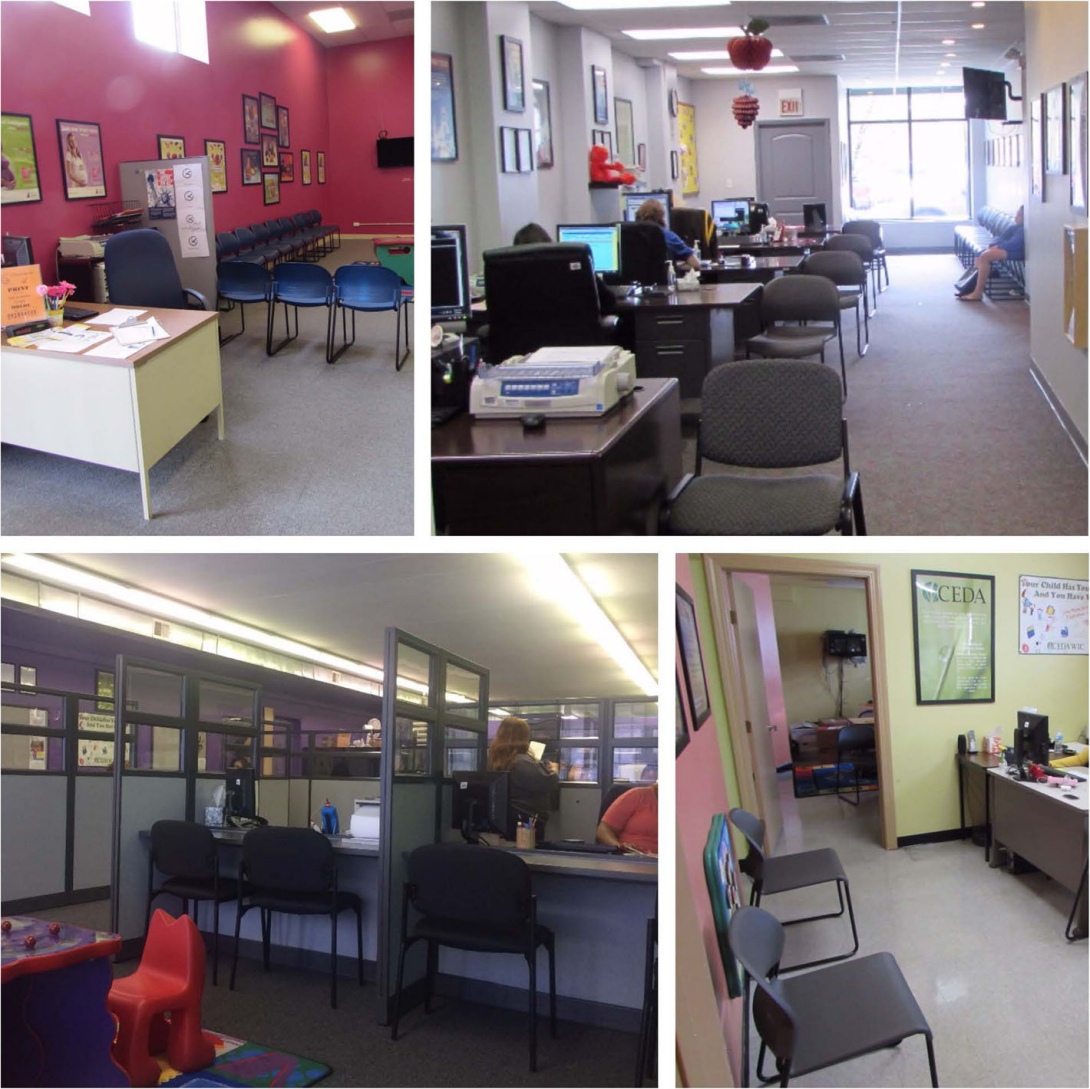
WIC centers: barriers to private space

The biggest feasibility issue with the proposed study design was physical space; several sites had no private space for data collection or intervention delivery. Figure 1 contains photographs of several WIC centers, and the spaces CHWs and research staff would have to work in. In some sites, CHWs or data collection staff would have to meet with families in full view of everyone else. Each site would require slightly different approaches for integrating CHWs, in order to account for site organization, provider styles, and ancillary site staff resources. Other implementation concerns identified by clinic and WIC center staff were related to how CHWs would integrate into the workflow. Staff were concerned that CHWs could delay their work and/or that interventions would lead to longer visits for families.

While the majority of caregivers and informants supported the plan for home visits from data collection staff and CHWs, important issues were identified. Families were nervous about the safety of letting unfamiliar people in the house and proposed getting to know staff in a neutral setting before letting them in the home. “For me it’s safety-wise. If you tell me, come to the meetings here and I already know her and I know she’s going to go to my house, oh, that’s fine and when are you going to go, Wednesday, and I’ll clean up, whatever, she can go, see you Wednesday, but if it’s just a random person, hi, I’m here from – I’ve never even seen you at a meeting. I don’t think I want to let you in. See that’s kind of my problem safety-wise.” (Hispanic female caregiver, English) Others discussed issues of poverty and having their home and lifestyle judged. “…I think the biggest fear I think for everybody is somebody going, kind of judging your house.” (Hispanic male caregiver, English)

The data produced a comprehensive picture of the family-level oral health resources, and barriers (see Additional file 1) that informed content areas the intervention should include. Families knew many places to get dental care but they struggled to find places that accepted public insurance and to pay for care not covered by insurance. They also felt Medicaid insurance resulted in worse quality of care than private insurance. Families demonstrated varying degrees of knowledge and comprehension of oral health behaviors. Major knowledge gaps were identified, especially regarding brushing equipment, fluoride, nutrition, and weaning. Fluoride exposure was limited, mainly because families do not drink fluoridated water. The complexity of oral health behaviors was a frequent observation. Weaning is an example of this. When asked if her child sleeps with a bottle, one mother said no but when prompted she followed with: “She just sleeps with it. She likes to hold it. She doesn’t sleep with it in her mouth or nothing. She just likes to hold it. She likes to sleep with it like a teddy bear or something.” (African-American female caregiver) Also, the beliefs and behaviors of family members directly influenced efforts to control behaviors of children, especially grandparents who are frequently the primary source of childcare. “… the mom goes to work and sometimes it’s the grandma who takes care of the child, so sometimes the mom will say, well, with me the child, I do not want to give the bottle anymore but when the child is with grandma, then grandma gives the bottle to the child…. ” (WIC center key informant, nutritionist technician); “I hear a lot mom wants to get the baby off the bottle or mom wants you know basically to wean them off the bottle but the dad doesn’t and says no, so just conflict.” (WIC center key informant, nutritionist) This suggests that support and education are needed for the entire extended family. Pain was not mentioned as a barrier to care; instead, being pain-free in the long term was the goal of caregivers for themselves and their children.

## Discussion

We conducted a comprehensive formative assessment in a high-risk population in Cook County, Illinois, to determine the feasibility and acceptability of a planned cluster-randomized controlled trial. Overall acceptance was high; families, clinics, and WICs generally expressed a need for oral health education for families of young children and approved of the CHW model. The results highlighted specific issues with the proposed implementation of this trial, leading to several critical design changes (Table 2).

**Table 2:** Changes in study design

Original study design	Challenge identified	Final study design
All data collected in homes	Some of the families identified as high-risk and most in need of the intervention were uncomfortable with letting someone into their home. Some clinics/WIC centers did not have sufficient space for data collection at the site.	Data collection in the home is still the primary goal, but we now also allow data collection to occur in the clinic/WIC centers or in another location (e.g., relative’s house).
Factorial design that included the following groups: - CHW in clinic only - CHW in WIC only - CHW in home only - CHW in clinic and home - CHW in WIC and home - No CHW	Families were less trusting of a CHW intervention that is not associated with a clinic or WIC center. Clinics and WIC centers expect CHWs to be flexible and meet families in settings outside of the clinic/WIC center	We simplified the design to a two-arm cluster randomized trial with wait-list control. One arm gets a CHW assigned to the clinic/WIC center, and the other receives CHW services when the study is over. CHWs work in the clinic/WIC and in homes, as preferred by families.
Oral health topics for CHW intervention chosen by investigators.	The specific needs of families did not align exactly with the topics chosen.	Oral health topics for CHW intervention were tailored to fit the specific needs of the families.

### Data collection feasibility

We had planned for all data collection to be conducted in the homes in order to best observe oral health behaviors in the environment they are performed. However, the potential for resistance to home visitation by high-risk families prompted us to revise this. While home data collection is still preferred, we opened the option for data collection to occur in the clinic/WIC center or in another location (e.g., relative’s house). This allows more high-risk families to engage in research and build trust with our staff, as well as accommodates the space issues of some of the sites.

### CHW intervention acceptability and feasibility

**Fig. 2:**
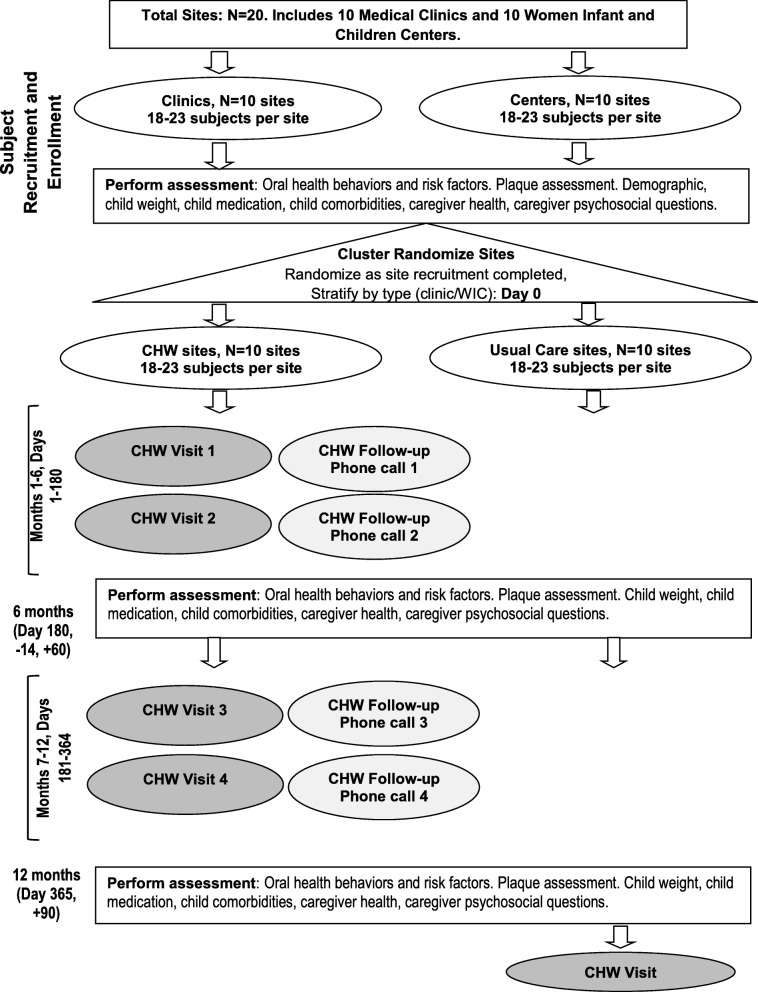
CO-OP Chicago final study design

We had originally intended to place oral health CHWs in three settings (medical clinics, WIC centers, and homes) and restrict them to only those settings to determine in which setting the model worked best. The results of the formative work suggested that families will be more trusting of a CHW intervention that is associated with a clinic or WIC center. Also, clinics and WIC centers expect CHWs to be flexible and meet families in settings outside of the clinic/WIC center. Linking CHWs with a trusted sustainable agency while also allowing them to work outside that agency has been described by others as a successful model [25–27]. Therefore, we eliminated the home-only arm of the trial. We also changed to allow the clinic and WIC center CHWs to meet families wherever they choose while using the clinic/WIC center as a home base. This design change had the consequence that some sites would not receive any intervention. We considered alternative implementation design approaches, including step-wedge, but the cluster randomized controlled trial design was ultimately determined to best fit the research aims. To ensure sites feel valued and engaged, we will provide them with things they asked for which include toothbrushes to give away, locally tailored referral lists of oral health providers, and educational tools. We also decided to offer participants at usual care sites the opportunity to have a CHW visit after the conclusion of the study. This ensures all participating sites at some point have access to oral health education and connection to community resources. The final study design is shown in Fig. 2.

### Intervention content tailoring

The oral health topics targeted by CHWs were matched to the needs of families. Five primary topics were identified: (1) nutrition (food security, sugary liquids with a focus on juice, healthy foods, how to plan for and encourage healthy foods, understanding the rationale for nutrition recommendations), (2) weaning (recommendations, strategies, the basics of sippy cups), (3) toothbrushing (when, how, equipment, establishing routines, toothpaste, spitting, wiping versus brushing, parent modeling, flossing), (4) fluoride (what it is and why it matters, water, varnish, toothpaste), and (5) access to dental services (where, insurance).

The oral health issues raised by informants and caregivers match the social determinants of oral health model by Fisher-Owens et al. [10] and emphasize the multi-level contributions to oral health disparities. Poverty was a big barrier mentioned by families and key informants. Limited access to proper dental care was another consistent theme. Medicaid eligibility restrictions and low reimbursement rates in Illinois reduce access to oral health providers for children and result in almost no affordable options for caregivers [28]. Children had dental coverage through Medicaid but still struggled to obtain quality affordable care. On the family level, the perceived importance of adequate oral health access to care and management routines for the entire family was a consistent theme. We heard caregiver confusion regarding the recommendations for oral health for children under the age of 3 years old, specifically with respect to toothbrushing, use of fluoridated toothpaste, and weaning. The persistence of these types of knowledge, skills, and access issues despite public health messaging and well-trained dental providers emphasize the need for alternative approaches such as CHWs [25, 26].

### Study limitations

This type of formative work has specific limitations. Our sample represents low-income neighborhoods in the Chicago area, and findings may not be applicable to smaller and rural communities, populations with different cultural traditions, and states or countries with different types of insurance and access to dental care. Because data that emerge from a specific social situation like a focus group or interview are highly contextualized and prone to social desirability, we used multiple sources of data to inform our results [29]. We merged standard research methodologies with elements from the field of design to capture a more comprehensive picture [22, 23, 30, 31].

## Conclusions

Behavioral intervention trials require major investments by participants, investigators, and funders. This manuscript describes efforts to prepare for a community-based trial testing an oral health intervention. The findings from this formative work demonstrated a perceived need for our proposed intervention and suggested design changes to improve study acceptance and feasibility. The next step is to test our oral health CHW intervention in a 400-subject cluster randomized controlled trial. While the direct results of our formative research may provide ideas for others conducting similar trials, the main conclusion is that this type of assessment is important to enhance trial success. Our final trial design incorporated input from the key trial stakeholders: clinics, WIC centers, and caregivers. The inclusion of all stakeholders and the use of multiple methodologies (interviews, focus groups, and observations) provided a comprehensive picture to guide study design and implementation. Others have found similar formative work that includes methodologies from the field of design to be highly effective in the planning of feasible sustainable interventions [32, 33]. While this type of formative work requires a significant upfront investment, we expect it will translate into savings through effective recruitment, retention, intervention implementation and adherence, and results dissemination. As we strive to improve the impact and efficiency of our research dollars, efforts such as this should be considered to maximize the success of behavioral trials in oral health and other areas.

## Additional file

Additional file 1: The information in the table is quotations from key informants and focus group participants about family strengths and challenges related to oral health. (DOCX 15 kb)

## Abbreviations

CHW: Community Health Workers
CO-OP: COordinated Oral health Promotion
WIC: Women Infant and Children

## References

[1] U.S. Department of Health and Human Services, National Institutes of Health, National Institute of Dental and Craniofacial Research (2017). Dental caries in children.

[2] Bader JD, Rozier GR, Lohr KN, Frame PS (2004). Physicians’ roles in preventing dental caries in preschool children: a summary of the evidence for the U.S. Preventive Task Force. Am J Prev Med.

[3] Casamassimo PS, Thikkurissy S, Edekstein BL, Maiorini E (2009). Beyond the human and economic cost of early childhood caries. J Am Dent Assoc.

[4] Kawashita Y, Kitamura M, Saito T. Early childhood caries. Int J Dent. 2011; Epub 2011 Oct 10. 10.1155/2011/725320 PMC3191784 22007218

[5] National Institute of Dental and Craniofacial Research, Dental Oral and Craniofacial Data Resource Center. Data tables for oral health indicators. Available at http://www.webcitation.org/getfile?fileid=e9d42542acaa10d972db27912c6c91da30060e1c. Accessed 28 Sept 2018.

[6] Schwendicke F, Dörfer CE, Schlattmann P, Page LF, Thomson WM, Paris S (2015). Socioeconomic inequality and caries: a systematic review and meta-analysis. J Dent Res.

[7] Illinois Department of Public Health (2017). Healthy smile healthy growth 2008–2009.

[8] Casamassimo P, Lee J, Marazita M, Milgrom P, Chi D, Divaris K (2014). Improving children’s oral health: an interdisciplinary research framework. J Dent Res.

[9] Segura A, Boulter S, Clark M, Gereige R, Krol DM, Mouradian W, Keels MA (2014). Maintaining and improving the oral health of young children. Pediatrics.

[10] Fisher-Owens SA, Gansky SA, Platt LJ, Weintraub JA, Soobader MJ, Bramlett MD (2007). Influences on children’s oral health: a conceptual model. Pediatrics.

[11] American Public Health Association Community Health Worker Section. Available at http://www.apha.org/apha-communities/member-sections/community-health-workers. Accessed 26 June 2017.

[12] U.S. Health Resources and Services Administration, Health Resources and Services Administration, Bureau of Health Professions. Community health workers national workforce study. Rockville: HRSA; 2007.

[13] Viswanathan M, Kraschnewski J, Nishikawa B, Morgan LC, Thieda P, Honeycutt A, Lohr KN, Jonas D. Outcomes of community health worker interventions. Evidence report/technology assessment no. 181 (prepared by the RTI International–University of North Carolina Evidence-based Practice Center under contract no. 290 2007 10056 I.) AHRQ publication no. 09-E014. Rockville: Agency for Healthcare Research and Quality; 2009.

[14] Viswanathan M, Kraschnewski JL, Nishikawa B, Morgan LC, Honeycutt AA, Thieda P, Lohr KN, Jonas DE (2010). Outcomes and costs of community health worker interventions: a systematic review. Med Care.

[15] Lewin SA, Dick J, Pond P, Zwarenstein M, Aja G, van Wyk B, Bosch-Capblanch X, Patrick M (2005). Lay health workers in primary and community health care. Cochrane Database Syst Rev.

[16] Rhodes SD, Foley KL, Zometa CS, Bloom FR (2007). Lay health advisor interventions among Hispanics/Latinos: a qualitative systematic review. Am J Prev Med.

[17] Feldens CA, Giugliani ER, Duncan BB, Drachler Mde L, Vítolo MR (2010). Long-term effectiveness of a nutritional program in reducing early childhood caries: a randomized trial. Community Dent Oral Epidemiol.

[18] Kowash MB, Pinfield A, Smith J, Curzon ME (2000). Effectiveness on oral health of a long-term health education programme for mothers with young children. Br Dent J.

[19] Plonka KA, Pukallus ML, Barnett A, Holcombe TF, Walsh LJ, Seow WK (2013). A controlled, longitudinal study of home visits compared to telephone contacts to prevent early childhood caries. Int J Pediatr Dent.

[20] Grover J (2014). The community dental health coordinator. A valued new member of the dental team. Todays FDA.

[21] A Statement from the American Dental Association (2012). Breaking down barriers to oral health for all Americans: the Community dental health coordinator.

[22] Beyer H, Holtzblatt K (1999). Contextual design. Interactions.

[23] Martin B, Hanington B (2012). Universal methods of design: 100 ways to research complex problems, develop innovative ideas and design effective solutions.

[24] Corbin J, Strauss A (2015). Basics of qualitative research: techniques and procedures for developing grounded theory.

[25] Gutierrez K, Campbell J (2014). Best practice guidelines for implementing and evaluating community health worker programs in health care settings.

[26] Rosenthal EL, Wiggins N, Brownstein JN, Johnson S, Borbon IA, De Zapien JG (1998). The final report of the National Community Health Advisor Study: weaving the future.

[27] Martin MA, Mosnaim GS, Olson D, Swider S, Karavolos K, Rothschild S (2015). Results from a community-based trial testing a community health worker asthma intervention in Puerto Rican youth in Chicago. J Asthma..

[28] Oral Health in Illinois. Medicaid-Participating Dentists. Available at: http://oralhealthillinois.org/data-and-maps/publicly-funded-services/participating-dentists. Accessed 23 June 2017.

[29] Sim J (1998). Collecting and analyzing qualitative data: issues raised by the focus group. J Adv Nurs.

[30] Glaser B, Strauss A (1967). The discovery of grounded theory: strategies for qualitative research.

[31] Corbin J, Strauss A (1990). Grounded theory research: procedures, canons, and evaluative criteria. Qual Sociol.

[32] Erwin K, Krishnan JA (2016). Redesigning healthcare to fit with people. BMJ.

[33] Martin MA, Press VG, Erwin K, Margellos-Anast H, Ignoffo S, McMahon KS, Mosnaim G, Ramsay J, Paik SM, CHICAGO Plan Investigators (2018). Engaging end-users in intervention research study design. J Asthma.

