# Supplementary material for: Formative research implications on design of a randomized controlled trial for oral health promotion in children
Source: Pilot Feasibility Stud. 2018 Oct 4;4:155. doi: 10.1186/s40814-018-0344-y (PMC6171134; doi:10.1186/s40814-018-0344-y)
Supplement: Supplementary file 1 — The information in the table is quotations from key informants and focus group participants about family strengths and challenges related to oral health. (DOCX 15 kb) [file 40814_2018_344_MOESM1_ESM.docx]

| **Family Strengths and Challenges Regarding Oral Health** | | |
| --- | --- | --- |
| **Final Themes** | **Representative Key**  **Informant Quotes** | **Representative Caregiver Quotes** |
| **Insurance and cost**: Families struggle to find places that accept their insurance and to pay for dental care that isn’t covered for their children or themselves. Families felt they received different, and inferior, service with Medicaid compared to private insurance. | "Well, definitely money. That’s a barrier, because most of their, like I said, most of our patients are Medicaid and if it doesn’t cover maybe braces or some other bigger procedure that they might need, then they have to pay out of pocket and most of our patients don’t have that type of money.”  "Yeah, it’s hard. Even with the insurance coverage a lot of our patients on Medicaid, a lot of services are not covered." | “(one speaker) It’s whatever HMO you got too, whatever that is. This dentist don’t take that. This dentist you can’t go there because of this, and that’s what the problem be too. (another speaker) And I kind of feel lost sometimes, like who do I call. Then I grab a number and they’re like, oh, we don’t take your insurance. (another speaker) Exactly, so now you got to run around, so now you getting discouraged because, oh, I thought I could go right up here when you got to go to Afghanistan to go to the dentist."  “…my son, he had a abscess in his mouth. I understand you have to come and you have to take the antibiotics first and the come back, but it’s like – when I had my job insurance, it was done in two visits. With public aid, it’s like done in four visits, so it’s like you constantly have to keep calling off work or rescheduling because they really don’t want to do it …” |
| **Fluoride sources:** Families do not understand fluoride recommendations and sources. Families do not get fluoride from city water because almost all drink bottled water. They drink bottled water because of habit, taste, and concerns for safety. | "They are bottled water drinkers. A lot of them drink bottled water. They don’t really drink that water out the faucet especially not over here. It’s not that the water is terrible in Harvey but I don’t know what’s going on in a lot of their homes, so most of them drink bottled water. All of my clients come in with bottles of water. They don’t drink the water out the faucet. " | “Because I see a lot of baby toothpaste have non-fluoride but then there’s fluoride in our water and there’s fluoride in other things that we use so it’s kind of confusing. Like, is it good or bad? "  "When I took my twins to the dentist, I told him I don’t drink lots of water because I don’t and so if I don’t buy bottled water then I ain’t drinking no water at all."  "Bottled water. That’s just a habit. Everybody go to the grocery store." |
| **Nutrition:** Families do not understand the risks of juices and other sugary drinks. They understand candy isn’t good for teeth. | “The only other thing I can think of and this might be too specific but with the sugary liquids, I know a lot of parents specifically with the Hispanic population are doing like the yogurt drinks, like the Yakult, I hear that a lot, or the Danimals, and I think they may not know how bad it is"  "I think actually a lot of families think like, well, it’s apple juice, it’s orange juice, it’s like healthy for them, and I don’t think they realize kind of the risk of the teeth. " | “Like if I give my daughter some juice or anything I still dilute it with water, because I don’t want her to be jumping off the wall. Like I get apple juice, orange juice, I just still put water up in it.”  “Like she gots cavities, lots of them, that’s because if she’s with my sister they let her eat candy all day nonstop so I don’t let my little ones do that.” |
| **Weaning:** Parents struggle to wean children from the bottle and from nighttime feedings because they do not understand the recommendations, they do not have the time or patience to enforce them, or because other caregivers do not support the efforts of the parents. | "The big challenge our clients have is getting their kids off the bottle. …, even if mom or dad are trying to get their child off the bottle, if they’re at grandma or grandpa’s house or an aunt or whatever."  "Um, I feel like barriers would be family members that think their children are still babies and maybe they don’t need to – you know, they’re so young, they don’t need to worry about their teeth yet or, you know, old-school practices that children are on bottles until 2 or 3 years old and maybe grandma was like, oh, they’re still a baby, they can have the bottle at night and bedtime and the mom wants to get them off but the other family members in the house – you know, maybe they’re babysitting and watching them more often than mom can, so they could have a negative influence." | "She just sleeps with it. She likes to hold it. She doesn’t sleep with it in her mouth or nothing. She just likes to hold it. She likes to sleep with it like a teddy bear or something. "  "Yo tengo un niño que duerme con, no duerme, se toma su teta antes de dormir. Pero en mamila y ya tiene cuatro años. Es malo eso?" [English translation: *I have a son that he sleeps with, he doesn’t sleep, he drinks from his bottle before going to sleep. But a baby bottle, and he is four years old. Is that bad?*]  “No, like I tried to take him off the bottle because the dentist told me to take him off because he going to be 2 next month, but he – we tried and I’m like, man, look just get on the bottle, I just don’t care. He’ll get his teeth fixed another time. I ain’t got time for this.”  “He’ll cry all day." |
| **Tooth brushing:** Families generally understand tooth brushing recommendations but they do not understand which brushes to use. They also struggle with child acceptance of brushing and with maintaining a brushing schedule due to busy lives. | " I think that they know that they should brush their children’s teeth at least twice a day, okay. So those are the tooth things that, you know, we have going for us. "  "Well most people usually when I tell them about brushing, have already a bought a toothbrush. That’s a good thing, even when kids don’t have teeth yet." | "What toothpaste to use. What toothbrush to use. Like I need for you to tell me like don’t say brush your teeth and then I go to the Walmart and there’s 50,000 different toothpastes. Tell me what I need to get. Now if it costs a little bit more I’m fine with that as long as I know that this is helping me. Like she said, she might buy something different from her. Me personally, toothpaste is toothpaste, whatever is free when I use a coupon, but if you tell me I have to get this particular brand toothpaste then that’s what I’m going to get. The toothbrush, all that, like the things that I need to do. And is mouthwash, is that like a necessity?"  "Yes, it’s a struggle. I even brought her the mouth wash that change the colors to see the plaque on the teeth to try to get different little ways to get her going, but it’s a struggle. They say twice a day but it’s hard to do even once. "  “Yeah, because you have like – if you have like a lot of children, you just have so much to do before it’s time to go to bed and so by the time you get done it’s like you just tired. You just want to go to sleep.” |
